# Beech Leaf Disease Severity Affects Ectomycorrhizal Colonization and Fungal Taxa Composition

**DOI:** 10.3390/jof9040497

**Published:** 2023-04-21

**Authors:** Claudia Bashian-Victoroff, Alexis Brown, Andrew L. Loyd, Sarah R. Carrino-Kyker, David J. Burke

**Affiliations:** 1 The Holden Arboretum, 9500 Sperry Road, Kirtland, OH 44094, USA; cvictoroff@holdenfg.org (C.B.-V.); brown.alexis365@gmail.com (A.B.); skyker@holdenfg.org (S.R.C.-K.); 2 Bartlett Tree Research Laboratories, 13768 Hamilton Rd., Charlotte, NC 28278, USA; aloyd@bartlett.com

**Keywords:** *Litylenchus crenatae*, fungal communities, ectomycorrhizal fungi, high-throughput sequencing, provenance

## Abstract

Beech leaf disease (BLD) is an emerging forest infestation affecting beech trees (*Fagus* spp.) in the midwestern and northeastern United States and southeastern Canada. BLD is attributed to the newly recognized nematode *Litylenchus crenatae* subsp. *mccannii*. First described in Lake County, Ohio, BLD leads to the disfigurement of leaves, canopy loss, and eventual tree mortality. Canopy loss limits photosynthetic capacity, likely impacting tree allocation to belowground carbon storage. Ectomycorrhizal fungi are root symbionts, which rely on the photosynthesis of autotrophs for nutrition and growth. Because BLD limits tree photosynthetic capacity, ECM fungi may receive less carbohydrates when associating with severely affected trees compared with trees without BLD symptoms. We sampled root fragments from cultivated *F. grandifolia* sourced from two provenances (Michigan and Maine) at two timepoints (fall 2020 and spring 2021) to test whether BLD symptom severity alters colonization by ectomycorrhizal fungi and fungal community composition. The studied trees are part of a long-term beech bark disease resistance plantation at the Holden Arboretum. We sampled from replicates across three levels of BLD symptom severity and compared fungal colonization via visual scoring of ectomycorrhizal root tip abundance. Effects of BLD on fungal communities were determined through high-throughput sequencing. We found that ectomycorrhizal root tip abundance was significantly reduced on the roots of individuals of the poor canopy condition resulting from BLD, but only in the fall 2020 collection. We found significantly more ectomycorrhizal root tips from root fragments collected in fall 2020 than in spring 2021, suggesting a seasonal effect. Community composition of ectomycorrhizal fungi was not impacted by tree condition but did vary between provenances. We found significant species level responses of ectomycorrhizal fungi between levels of both provenance and tree condition. Of the taxa analyzed, two zOTUs had significantly lower abundance in high-symptomatology trees compared with low-symptomatology trees. These results provide the first indication of a belowground effect of BLD on ectomycorrhizal fungi and contribute further evidence to the role of these root symbionts in studies of tree disease and forest pathology.

## 1. Introduction

Symptoms of beech leaf disease (BLD) were first described in 2012 in Lake County, Ohio [1]. BLD is attributed to the newly recognized nematode *Litylenchus crenatae* subsp. *mccannii* [2]. Although other bacteria or fungi may be involved in BLD, either as nematode symbionts or as opportunistic pathogens [3], *Litylenchus crenatae* ssp. *mccannii* is required for BLD development [2]. Early evidence of BLD infestation can be identified through a combination of canopy symptoms including the darkening and thickening of leaves and the appearance of interveinal banding [1,3]. Eventually, BLD infestation leads to canopy senescence and tree mortality [1]. The presence of BLD has been described in forests from Ohio and southern Ontario, across the eastern United States and Canada, and into New York and New England (see https://www.clevelandmetroparks.com/parks/education/publications/ (accessed on 22 March 2023); [4]). Beech leaf disease also affects a number of different *Fagus* species, including American (*Fagus grandifolia*), European (*F. sylvatica*), Chinese (*F. engleriana*), and Oriental (*F. orientalis*) [3]. While BLD affects a range of *Fagus* species, of greatest concern from a forest conservation standpoint in North America is the mortality of *F. grandifolia*. *F. grandifolia* is a native, dominant canopy species in many forests in the U.S. and Canada. *F. grandifolia* is characteristic of midwestern forests in the Great Lakes region, currently making up >80% of overstory basal area in Beech–Maple–Birch dominated forests, according to the USDA Forest Service Forest Inventory Assessment [5]. The emergence of BLD presents a major threat to the survival and persistence of beech trees across North America [4].

Reductions in leaf survival and canopy extent from BLD may also affect other organisms that rely upon beech trees for resources, especially root-associated fungi that form mycorrhizal associations. Mycorrhizal fungi form symbiotic relationships with plant roots, and provide plants with improved access to soil resources such as nitrogen, phosphorus, and water [6]. Through unique enzymatic processes, mycorrhiza-forming fungi absorb a portion of the plant’s photosynthetically derived carbohydrates at the interface of plant root cells and fungal hyphae [7]. Mycorrhizal fungi rely almost entirely on photosynthate produced by plant partners to grow and function [8], though some species also produce carbon mobilizing enzymes [9]. Ectomycorrhizal (ECM) fungi colonize the roots of many temperate hardwood trees, including *Fagus* spp., and are characterized by the morphology of mycorrhizal structures, determined in most cases by the plant partner [10,11]. In addition to aiding plant nutrient acquisition, ECM is associated with improved stress tolerance [12], plant drought tolerance [13], and resistance to soil pathogens [14].

Phytophagous insects, which can reduce leaf area, and wood boring insects, which can disrupt and reduce the flow of photosynthetically derived carbohydrates to roots, can negatively affect mycorrhizal fungi [15,16]. For example, in stands of eastern hemlock (*Tsuga canadensis*) experiencing infestation by the invasive phytophagous insect hemlock woolly adelgid (*Adelges tsugae*), infestation decreased the percentage of roots colonized by ECM fungi by over 67% [17] and decreased ECM inoculum potential in soil, impeding forest recovery of lost trees [18]. In studies of pine wilt disease (PWD), which is caused by the pine wilt nematode (*Bursaphelenchus xylophilus*), diseased trees reduced ECM fungal colonization, along with lower richness and diversity, and community structure shifts of root-associated fungi, including ECM fungi [19,20]. Beech bark disease (BBD), which is the result of co-infestation by the beech scale insect (*Cryptococcus fagisuga*) and *Neonectria* fungal pathogens, can ultimately girdle diseased trees [21]. Tree girdling also affects the diversity of ECM fungi and mycorrhizal function [22]. BLD reductions in ECM colonization could further negatively impact tree health if reductions lead to impaired tree nutrient uptake in the diseased state. Further, because of the role ECM fungi have on carbon and nutrient cycling [23], the impact of tree disease on these root symbionts can have cascading effects on forest function overall. To date, however, the effect of BLD on ECM fungi colonizing infested *F. grandifolia* trees is unknown.

This research investigates the colonization and community structure of ECM fungi on roots of American beech displaying varying levels of BLD symptomatology. Our objective was to determine if the severity of BLD symptoms and leaf damage is associated with differences in ECM fungi belowground. We aimed to accomplish this objective by the following: (1) qualitatively ranking BLD conditions based on visual symptoms into three categories, (2) quantifying and comparing ECM fungal root tip colonization between each category, and (3) describing ECM community structure in each tree condition level. Because ECM fungi rely on the photosynthate of their autotrophic hosts, and because BLD affects tree photosynthetic capacity through leaf senescence and canopy decline, we hypothesized that trees in poor condition would have significantly lower ECM colonization than healthy trees. Further, because ECM fungi are functionally divergent in their host carbon demand and symbiotic roles [24,25], we hypothesized that ECM community composition would vary across disease conditions. Finally, because mycorrhizal partnerships may be locally adapted [26,27], we hypothesized that tree provenance would impact fungal community composition.

## 2. Methods

### 2.1. Site Description

Our study site is a beech bark disease resistance plantation established in 2006 and located at the Holden Arboretum in Kirtland, Ohio, USA [28]. All trees used within this study are part of an ongoing common garden experiment in which *F. grandifolia* were selected for and show resistance to beech bark disease [29] (Table 1, Figure S1). All individuals were grown from seed in their source location and accessioned to the Holden Arboretum as plants. Trees used in this study represent trees from two provenances (Maine and Michigan) that were planted at the Holden Arboretum in 2006 (Figure S1). At the time of the study, trees ranged from approximately 19 to 20 years old (ranging from 12 cm to 24 cm, DBH). In September of 2020, we qualitatively rated each of the beech trees based on symptoms of BLD. Ratings were based on a calculated symptom severity score. Trees were ranked by two observers, each of whom estimated the percentage of symptomatic leaves at three locations around the tree. Rankings were given a numerical value based on these percentages (Table S1). These values were averaged to a whole number and were used to determine the ultimate symptomatology category of the tree (high, intermediate, and low symptomatology).

### 2.2. Sampling Methods

Representative trees of each condition level (high symptomatology—trees of poor condition, intermediate symptomatology—some evidence of BLD, and low symptomatology—little to no evidence of BLD) were selected for sampling across two sampling efforts in October 2020 and June 2021 (Table 1, Figure S1). In October (fall) 2020, we sampled root sections of 26 trees with varied symptoms of BLD at three different locations around the selected tree with each of the three samples approximately 50 cm from the base of each tree. Roots were exposed gently with a hand shovel and roots up to 1 cm in diameter were excised with pruning shears and placed into a sterile bag. Root sections from each tree were pooled into one sample representing that tree. In June (spring) 2021, we repeated sampling of root fragments from the same individuals as well as four additional individuals to better replicate the Maine provenance.

### 2.3. Root Processing

We gently rinsed root fragments over a sieve to remove rhizosphere soil. Under a magnifying lens, we initially isolated fine root tips from coarse root sections. Fine root tips were further examined under a dissecting scope at 10–40× magnification to determine whether tips were (1) alive or dead and (2) colonized by ECM fungi. Live ECM fungi were counted and separated for further community analysis using molecular methods. Roots found to either be dead or not colonized by ECM fungi were discarded. We dried and weighed the remaining coarse root sections to standardize the number of living root tips based on the mass of roots collected. Root colonization data are represented as the number of colonized ECM fungal root tips per gram of woody root biomass.

### 2.4. Molecular Methods

Fungal DNA was extracted from an approximately 0.5 g subsample of the isolated root tips from each of the 56 root samples (from 26 trees in October 2020 and 30 trees in June 2021). We utilized a combination of liquid nitrogen and bead beating to pulverize the root tips. DNA was purified using phenol–chloroform extraction [30] and was precipitated using 20% polyethylene glycol 8000, incubated, and desalted using 80% EtOH. Precipitated DNA was dried and resuspended in 50 μL TE (Tris EDTA) buffer and stored in a 1.5 mL low-retention microcentrifuge tube at −20 °C until it was used for PCR. In the polymerase chain reaction, we targeted the internal transcribe tracer (ITS) 2 region using the 58A2F (TCGTCGGCAGCGTCAGATGTGTATAAGAGACAG ATCGATGAAGAACGCAG) and ITS4 (GTCTCGTGGGCTCGGAGATGTGTATAAGAGACAG TCCTCCGCTTATTGATATGC) primers [31] containing Illumina overhang adapters (underlined portions). The ITS2 region was chosen for sequencing, as it has been shown to be an accurate marker for fungal diversity and taxonomic assignment, consistent with sequencing of the entire fungal ITS region (including both ITS1 and ITS2; [32]). Amplicons were sent to the Case Western Reserve University Genomics Core Laboratory (Cleveland, OH, USA), where multiplexing indices and Illumina sequencing adapters were added to the amplicons. The amplicons were then pooled and sequenced on a 2 × 250 bp high-throughput sequencing run using the Illumina Mi-Seq V3 sequencer (Illumina Inc., San Diego, CA, USA). For quality control, negative controls were included in our DNA extraction and PCR steps to ensure no outside contamination of fungal DNA, and a mock community of fungi (provided by Loyd, personal communication) was included on the sequencing run to ensure adequate coverage by our bioinformatics pipeline (details of the pipeline are in the following section).

### 2.5. Pipeline

Sample 57-M, which is a tree exhibiting high symptomatology sourced from the Michigan provenance, produced less than 500 sequence reads in the Fall 2020 collection and was thus removed from the analysis. Sequence reads from the remaining 55 root samples (sequence reads ranged from 2522 to 58,957 reads per sample) were processed with the UNOISE3 pipeline [33]. Briefly, forward and reverse sequence reads were merged with the fastq_mergepairs command in USEARCH version 11.0.667 [34] and control phiX reads were removed prior to merging reads using the filter_phiX command. Reads were trimmed of PCR primers using Cut Adapt (v2.8; [35]), where up to 15% mismatches were allowed during primer removal. Reads less than 300 bp in length and with one or more sequence errors were removed with the fastq_filter command. Denoising was performed with the unoise3 command, which resulted in error-corrected and chimera-filtered sequence variants (i.e., zero radius OTUs or zOTUs). The unoise3 command removed chimeras as well as zOTUs with fewer than 8 sequence reads (per the default settings). The merged reads with control phiX and primers removed were then mapped to the zOTUs with the otutab command. We made taxonomic assignments for each zOTU with the SINTAX algorithm [36] by comparing against the UNITE database (v 8.0, release date 18 November 2018; [37]).

### 2.6. Statistical Analysis

#### 2.6.1. ECM Root Colonization Analysis

Mean root tip abundance across each level of the categorical variable ‘Condition’ were compared using the function tapply in Base R [38]. The data distribution was visualized on a histogram, and abundance values were log transformed to improve normality of the variance. We used a generalized least squares (gls) model in the R package nlme to analyze whether ECM root tip colonization varied according to tree disease condition and based on tree provenance [39]. Root tip abundances from both collections were plotted for each level of tree condition to compare symptomatology effects between seasons, and seasonal abundances were plotted for each provenance to compare tree source effects and seasonality. Plotting was achieved using ggplot2 (v. 3.3.6; [40]).

#### 2.6.2. High-Throughput Sequence Analysis

We first utilized the Bioconductor package *phyloseq* to visualize fungal community results from high-throughput sequencing (v. 1.34.0; [41]). To visualize and compare the fungal communities across provenance and tree condition, we plotted the relative abundance of the 15 most prevalent fungal genera in stacked bar graphs using *phyloseq* (v. 1.40.0) and *ggplot2* (v. 3.3.6; [40]). To visualize fungal community structuring, we utilized Principal Coordinates Analysis (PCoA) using plotting functions in the *phyloseq* package. We applied the Sorenson (Bray–Curtis) distance metric to map the data onto a two-dimensional plot using the ordinate function. Separate plots were created for each collection (fall 2020 and spring 2021) to better visualize community structuring. We analyzed differences in fungal community composition from different tree conditions and between provenances using permutational multivariate analysis of variance (PERMANOVA) in the R package *vegan* (v.2.6.2; [42]). We used a two-way PERMANOVA on a distance matrix formed using the vegdist function in vegan to test the interaction between tree condition and provenance [42]. We used two additional one-way models to analyze the effect of tree condition on fungal community composition within each provenance. We ran additional one-way PERMANOVA models to analyze the effect of provenance and tree condition on distance matrices from the fall and spring collections independently. We analyzed the homogeneity of variances between levels for season, symptomatology, and provenance with permutational multivariate analysis of dispersion (PERMDISP). For this, we used the betadisper function in vegan and analyzed variance between beta dispersion using ANOVA. Alpha diversity metrics were calculated in *vegan* and included Shannon diversity (H), Simpson’s diversity (1-D), and Inverse Simpson’s (1/D). The fungal community data used in PCoA, PERMANOVA, PERMDISP, and alpha diversity calculations were normalized to control for variability in the number of sequences per sample (McMurdie and Holmes, 2014). For this, normalized sequence counts were calculated with the estimateSizeFactors function in the *DESeq2* package (v. 1.36.0; [43]).

#### 2.6.3. Wald Tests

To determine how fungal identities shifted at the zOTU level in different conditions and between provenances, we ran Wald tests in the *DESeq2* package with the function DESeq and included the alpha = 0.05 option. Three separate Wald tests were run; the first one compared Maine and Michigan trees, and the latter two compared trees of both high and intermediate symptomatology to trees of low symptomatology. For these tests, the Maine trees or low-symptomatology trees were set as the baseline in the contrasts. zOTUs were considered significantly different between tree provenance or condition if the adjusted *p*-values, implemented by default in *DESeq2* with the Benjamini–Hochberg false discovery rate, were below 0.05. In addition, the logarithmic fold change (LFC) values reported here were adjusted to account for low sequence numbers and large dispersions with the lfcShrink function in *DESeq2,* which used type = apeglm from the *apeglm* package (v. 1.18.0), as this method has been shown to have less bias than the default [44]. For each significant zOTU, plots of the normalized counts between provenance or condition were generated with the plotCounts function in *DESeq2* and one outlying point was removed that was driving a change in the direction of the LFC value between provenances once shrunk. Significant zOTUs were also assigned their potential ecological role with the FUNGuild Database (Nguyen et al., 2016) at the genus level or lowest taxonomic identity available if the zOTU could not be assigned to a genus or if the genus did not match a guild within FUNGuild. While some fungal taxa fall into multiple guilds and their function can depend on resource availability and life stage [45], the classification of taxa into functional guilds allows for, at least, the potential functioning of fungi to be explored.

## 3. Results

### 3.1. Root Colonization by ECM Fungi

**Figure 1:**
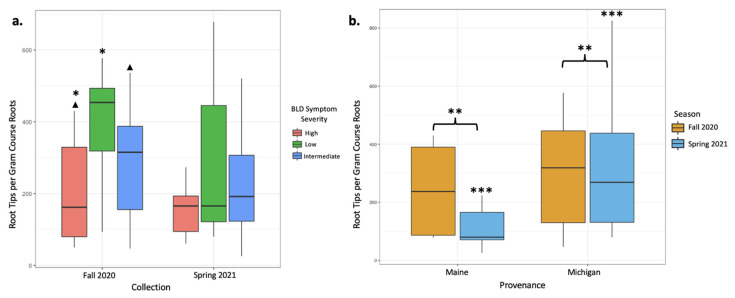
Mycorrhizal colonization expressed as root tip abundances per gram of dried course root material was plotted to indicate significant differences between treatments. (**a**) Colonization is plotted according to BLD symptom severity in both the fall 2020 and spring 2021 collections. The fall 2020 collection showed significantly different abundances between high- and low-symptom severity trees (*p* = 0.03; *) and moderate differences between high- and intermediate-symptom severity trees (*p* = 0.07; ▲). (**b**) Colonization is plotted according to collection season for both provenances (Maine and Michigan) to visualize differences in colonization according to tree provenance. Michigan trees had significantly greater colonization regardless of season, as indicated by root tip abundance (*p* = 0.003; **). While provenance did not impact colonization in the fall collection alone, colonizations from the spring collection were significantly different between Maine and Michigan trees (*p* < 0.001, ***).

In total, between our two sampling efforts, we counted 72,338 ECM root tips, 50,239 of which were counted from 26 sampled trees in the fall of 2020 and 22,399 were counted from 30 trees sampled in the spring of 2021. Based on tip abundance, mycorrhizal colonization varied broadly between samples. In the fall 2020 root collection, mycorrhizal colonization was highest in low-symptomatology trees (an average of 549.6 colonized root tips per g of dried root; Table 2). Intermediate-symptomatology trees had a 29.3% reduction in mycorrhizal colonization compared with low-symptomatology trees (an average of 388.7 colonized root tips per g of dried root; Table 2). Trees with high symptoms of BLD had a 65.7% reduction in mycorrhizal colonization compared with low-symptomatology trees (an average of 188.6 colonized root tips per g of dried root; Table 2). ECM colonization rates significantly varied between trees of low and high symptomatology (*p* = 0.03; Table 3, Figure 1a), and moderately varied between trees of high and intermediate symptomatology (*p* = 0.07; Table 3, Figure 1a). Data from the June 2021 root collections, however, yielded different results. In the spring collection, trees with varying levels of BLD symptoms did not have statistically different numbers of colonized root tips (Table 2 and Table 3). When data were averaged across both seasons’ collections, root tip abundance did not respond significantly to tree symptomatology, despite a 41.8% reduction in mycorrhizal colonization in high-symptom trees compared with low-symptom trees and a 13.8% reduction in mycorrhizal colonization in intermediate-symptom trees compared with low-symptom trees. This lack of significant change is likely due to large variations in root tip abundances between samples and collections (Table 2, Figure 1b).

Overall, mycorrhizal colonization was reduced in the spring 2021 collection by 29% relative to the fall 2020 collection, and these differences appear to be driven by spring effects on the Maine trees. Tree provenance (Maine- versus Michigan-sourced trees) yielded different numbers of colonized root tips. The mean mycorrhizal colonization from Michigan trees was three times greater than that from Maine trees when data from both collections were included in the analysis (*p* < 0.001, Figure 1b, Table 4 and Table 5). When data were subset by season, differences in root tip abundances in Maine and Michigan trees were significant in the spring collections, with Michigan trees having 65.4% more root tips per gram of dry roots than Maine trees (*p* = 0.003, Figure 1b, Table 4 and Table 5), but not in the fall collection (Table 4 and Table 5). Maine trees had 53.1% fewer root tips in the spring collection than the fall collection, whereas Michigan trees remain rather consistent in terms of root tip abundance (Figure 1b).

### 3.2. Community Composition and Diversity of Root Fungi

**Figure 2:**
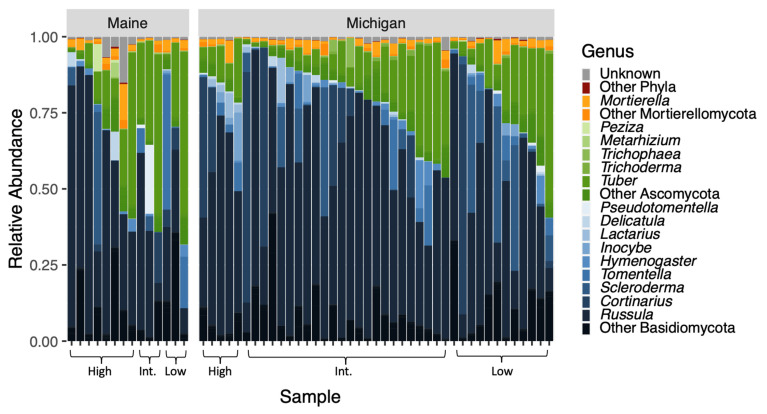
The relative abundances of OTUs of fungal genera within the three most prominent phyla are plotted according to tree provenance and BLD symptom severity. In this plot, phyla are represented by different colors and genera within those phyla are shades of those colors. Our sequencing effort yielded 1,178,687 reads across 677 OTUs. Analysis of community composition elucidated that fungi were significantly different between the tree provenances (*F* = 2.1538; *p* = 0.0074).

Our sequencing effort yielded 1,178,687 reads across 677 OTUs. Seven phyla were represented, including Ascomycota (367/677 OTUs; approximately 54%), Basidiomycota (216/677 OTUs; approximately 32%), and Mortierellomycota (36/677 OTUs; approximately 5%) (Figure 2). Just over 7% of OTUs (49/677) were unclassified fungi. Few representatives existed outside of the major ECM-forming phyla and were likely contaminants on living root tips. Although we extracted data from washed ECM root tips, sequencing yielded taxa in functional groups beyond ECM fungi.

**Figure 3:**
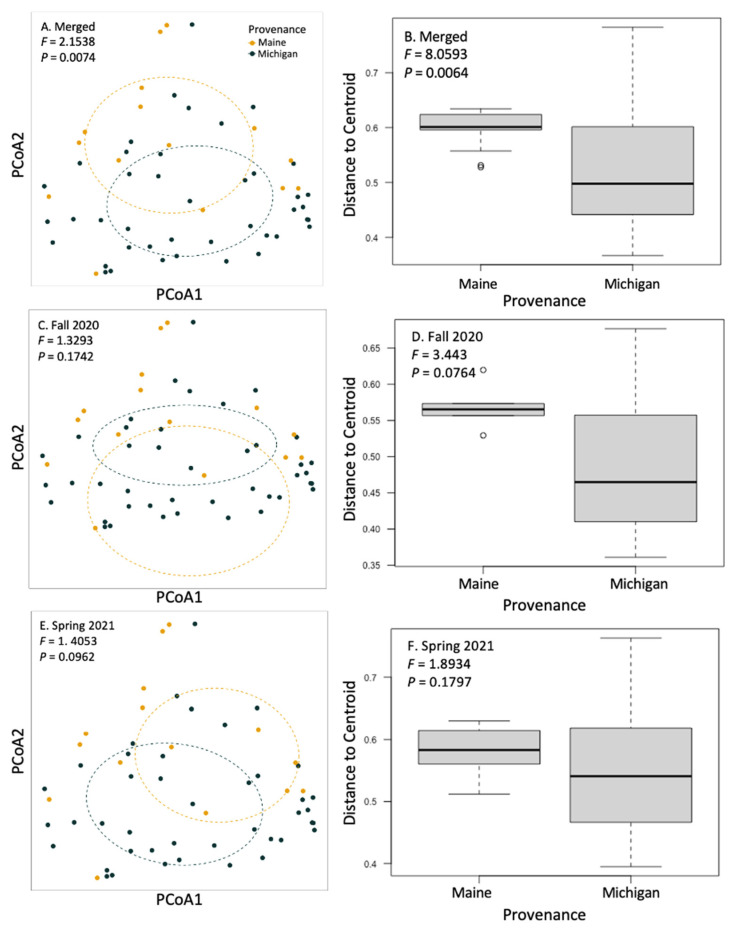
Principal coordinate analysis and beta dispersal of communities of fungi on the roots of sampled trees. We analyzed the effect of tree provenance on fungal community structuring and homogeneity of group dispersions. Fungal community composition did not respond to BLD symptomatology but did respond to tree provenance (*F* = 2.1538; *p* = 0.0074) when fall 2020 and spring 2021 data were merged (**A**,**B**). When trees from each collection (fall 2020 and spring 2021) were analyzed alone, provenance differences trended towards significance, as indicated by PERMANOVA or PERMDISP (**C**–**F**).

**Figure 4:**
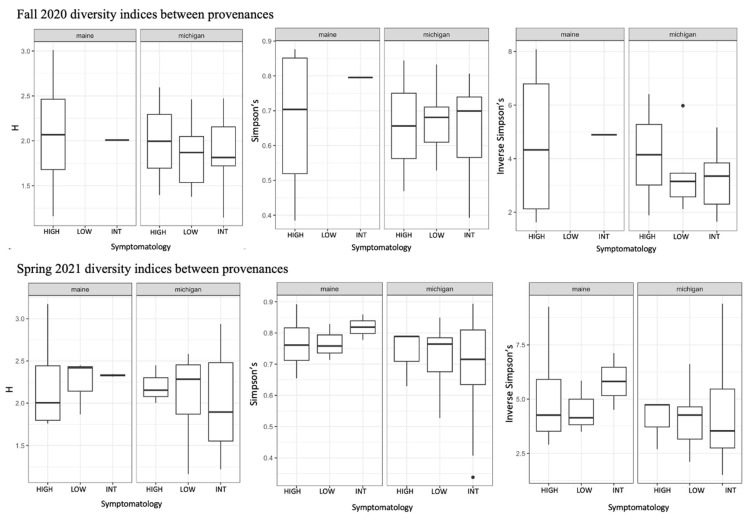
Results of diversity indices comparing root fungi from high, intermediate, and low symptom trees in each province for both collection periods. We analyzed Shannon diversity (H), Simpson’s diversity, and Inverse Simpson’s diversity between all groups (provenance, collection season, and tree symptomatology. In no instances were there significant differences in alpha diversity between levels within each group.

We used a community matrix of zOTUs and *DESeq2* normalized abundances to analyze differences in community composition and structure in different provenances and levels of symptomatology. Fungal community composition was significantly impacted by tree provenance. Provenance (trees sources from Maine verses Michigan) caused fungal community composition and community homogeneity to vary when both seasons’ collections were analyzed together (Figure 3A,B; *F* = 2.1538, *p* = 0.0074). When fungal communities from each season (fall 2020 and spring 2021) were analyzed independently, the signal was not significant (Figure 3C–F). Collection season and BLD symptomatology did not cause community compositional differences in root fungi. There were no significant differences in alpha diversity between different groups including tree provenance, collection season, and BLD symptomatology (Figure 4).

### 3.3. Wald Tests of zOTU Abundance by Provenance and BLD Symptomatology

Although the fungal community did not shift based on BLD symptomatology, our analysis yielded individual species differences based on BLD symptom severity. While mycorrhizal colonization was significantly lower in high-symptomatology trees relative to low-symptomatology trees, some individual zOTUs had greater normalized sequence abundance on trees with high or intermediate BLD symptom severity when compared with trees with low BLD symptom severity. These include zOTUs in the ECM or presumed ECM genera *Tuber*, *Russula*, *Cortinarius*, *Lactarius,* and *Hympenogaster;* in pathotrophic or possibly pathotrophic families *Nectriaceae* (including the genus *Fusarium*) and *Herpotrichiellaceae* (including *Minimelanolocus*); and others in orders Agaricales and Chaetothyriales, and phylum Ascomycota (Table S2). Two zOTUs that matched in the family *Herpotrichiellaceae* and order Agaricales were in lower abundance in high-symptomatology trees compared with low-symptomatology trees (Table S2). On average, the zOTU in Agaricales was reduced by 0.59% in high- relative to low-symptomatology plots and the zOTU in *Herpotrichiellaceae* was reduced by 0.17% in high- relative to low-symptomatology plots. Community compositional shifts between provenance may have been driven by a handful of individual species, which tended to be significantly more abundant on the roots of Michigan trees than Maine trees. This result corresponds with higher root tip abundances overall from Michigan trees. We found significantly higher normalized abundances in Michigan compared with Maine trees from species in the ECM genera *Lactarius*, *Tuber*, and *Cortinarius*; species in the saprotrophic genera *Mortierella* and *Gymnopus,* and the possibly pathotrophic genus *Minimelanolocus;* and others in orders Agaricales and Chaetothyriales, as well as phylum Basidiomycota (Table S2). One zOTU that matched with the genus *Tuber* was in lower normalized abundance in Michigan compared with Maine trees (Table S2).

## 4. Discussion

Beech leaf disease is an emerging threat to temperate forests in North America. Over time, BLD infestation leads to leaf senescence and canopy loss. Tree death, especially in the subcanopy, is common. Since ECM fungi rely on host allocation of photosynthetically derived carbohydrates to roots, leaf senescence and canopy loss may have consequences for root symbionts. We hypothesized that (1) trees more severely affected by BLD leaf symptoms would have lower ECM root colonization compared with trees less affected by BLD; (2) trees with different BLD symptom severity would host different fungal communities; and (3) because mycorrhizal partnerships are locally adapted, tree provenance would impact ECM root colonization and fungal community composition. Our predictions that ECM colonization and fungal communities would be affected by BLD symptom severity, and that plant provenance would also have a significant effect, were supported by our data.

Because fungi rely on the photosynthate of autotrophic hosts [46], and because BLD causes canopy loss, likely hindering photosynthesis [47], we predicted that trees more severely affected by BLD symptoms would have lower ECM root colonization. Colonized root tip abundance during fall sampling varied significantly between high BLD symptom severity trees and low BLD symptom severity trees, supporting our first hypothesis. Mycorrhizal colonization was almost three times greater (65.7%) in trees with low symptomatology than in those with high symptomatology. Root colonization varied widely in trees of intermediate symptom severity, with insignificant differences between this category and trees with low or high symptom severity. These results are similar to those described in eastern hemlocks, another ECM host tree experiencing canopy die-off due to an invasive phytophagous insect [17]. However, this pattern of reduced root colonization in highly symptomatic trees was evident only for fall root collections, as we found no significant effects of tree condition on root tip abundance from the spring sampling. This difference in response appeared to be driven in part by declines in root colonization on trees with intermediate and low symptomatology from fall to spring sampling, whereas high-symptomatology trees showed little change in root colonization between seasons. Seasonal changes in mycorrhizal root colonization and fungal communities have been observed in previous studies [48,49], and fine root growth often increases in the warmest months of the growing season [50]. Higher levels of root biomass and ECM colonization might therefore be expected in fall, and this was the case in the trees with intermediate and low BLD symptom severity. The response of trees severely affected by BLD in fall may be related to increased leaf mortality and overall reduced photosynthetic capacity of the trees throughout the growing season, leading to reduced summer root growth.

Despite changes in root colonization, we did not observe differences in root fungal communities with severity of BLD, which contradicted our second hypothesis. In previous studies, fungal community composition has shifted in response to tree pathology [19,20], environmental stress [51], and soil chemistry changes [25,52,53]. We predicted that functional and morphological divergence between groups of ECM fungi would lead some groups to be disproportionately impacted by disease severity. We did observe some individual zOTU level responses to disease symptom severity, as indicated by the Wald test, but the direction of those responses was different than anticipated. When we analyzed zOTU abundance at each level of disease severity, we predominantly found zOTUs that were significantly more abundant on the roots of high- and intermediate-symptomatology trees relative to low-symptomatology trees. While ECM fungal colonization was suppressed overall (as indicated by root tip abundances), some species—especially members in the genera *Tuber*, *Russula*, *Lactarius,* and *Cortinarius*—seem to have increased in normalized sequence abundance with increased disease severity. Species in the *Russulaceae* tend to have low-contact-type hyphae with no rhizomorphs [24,25], potentially indicating a lower carbon draw from plant hosts. If BLD impacts the photosynthetic capacity of beech trees, highly symptomatic trees may form preferential associations with low-carbon-demand fungi, which could explain the observed increase in low-carbon morphologies. Generally, little is known about special patterns of hyphal exploration among *Tuber* spp. [54], but some species have been indicated as contact type hyphae, such as *Russulaceae* [25]. *Cortinarius*, however, is indicated as having medium distance fringe or mat-exploration-type hyphae with rhizomorphs, potentially exerting a greater carbon demand on the host tree [25].

For our third hypothesis, we predicted that because mycorrhizal partnerships are locally adapted, tree provenance would impact ECM root colonization and fungal community composition. Our data supported this hypothesis in regard to both ECM colonization and fungal community composition. Past work has demonstrated that, due to local adaptations, tree provenance is an important factor in tree/fungal compatibility to form mycorrhizae [27]. When we compared our results between trees of different provenances, we found that the root-associated fungi on Michigan and Maine trees formed different community structures when data from both the fall 2020 and spring 2021 collections were combined (Figure 3). These community level differences may be driven by zOTUs, which were significantly more abundant on the roots of Michigan trees than on those from Maine. We found higher abundances from species in the ECM genera *Lactarius*, *Tuber*, and *Cortinarius*; species in the saprotrophic genera *Mortierella* and *Gymnopus,* and the possibly pathotrophic genus *Minimelanolocus;* and others in orders Agaricales and Chaetothyriales, as well as phylum Basidiomycota. This result coincides with overall higher ECM colonization in Michigan trees. Trees from the Michigan provenance may have greater compatibility with naturally occurring ECM fungi in Ohio soils than those from Maine due to relative geographic proximity to our Ohio field site. Reciprocal cross-inoculation experiments with arbuscular mycorrhizal (AM) fungi support that mycorrhizal fungi may be locally adapted to the conditions of their local soils [26] and, further, that plant hosts may harbor more mycorrhizae when grown with sympatric AM fungi than with allopatric AM fungi [26,55]. ECM fungi, similarly, may be adapted to local genotypes of host trees, as evidenced through reciprocal cross-inoculation leading to greater compatibility in sympatric combinations of plant genotype and ECM fungal community [56]. There is yet insufficient information on local adaptation in the ECM system to analyze these effects through meta-analysis [57].

In addition to providing host trees access to necessary soil nutrients, ECM may be functionally important in plant disease resistance [58] to common soil pathogens (Chu et al., 2019) as well as to infestations by phytophagous and wood-boring insects [59,60,61]. In the instance of infestations caused by phytophagous and wood-boring insects, ECM fungi may offer increased resistance to the insects, either through improved plant nutrition, induced systemic resistance [59,60,61], or cultivations of other beneficial organisms in the rhizosphere (e.g., Pine Wilt Disease; [62]). Our data largely lacked zOTUs, which were significantly more abundant at low disease severity compared with high or intermediate disease severity (Table S2). A lack of significantly greater abundance of any zOTU on trees of low symptomatology suggests that specific ECM fungal taxa are not functioning to reduce BLD symptom severity through the above mechanisms, though further work is needed to confirm this conclusion. On the other hand, however, the reduction in ECM root tips in high BLD symptomology relative to low BLD symptomology trees suggests that there is an effect of BLD severity on the mycorrhizal dynamics of beech.

In conclusion, we found evidence that the severity of BLD symptoms on *F. grandifolia* grown in a common garden experiment significantly reduced ECM colonization and had some specific effects on the occurrence of ECM taxa, although overall communities were unaffected by BLD. ECM provides a suite of ecosystem services [63] that aid plant nutrition and resilience, without which tree and ecosystem health would suffer. The loss of ECM symbionts, and the loss of nutrients coincident with ECM reduction, could have knock-on effects on trees suffering from BLD where canopy loss from nematode feeding is accompanied by nutrient uptake reduction from loss of ECM fungi, further negatively affecting tree health. Furthermore, if the inoculum potential of ECM fungi is reduced in forest settings that are affected by BLD, reductions in the biodiversity of ECM fungi could cause reductions in beech regeneration in the years to come. In this study, because root samples were collected from beech trees planted in a monoculture, it is unlikely that ECM fungi were able to supplement their carbon uptake from alternative hosts (e.g., other trees of the *Fagaceae* such as the genus *Quercus*), potentially amplifying the effect of BLD on ECM root tip abundance [64]. Subsequent efforts may clarify if these results would reoccur if roots were collected from trees in a mixed stand. We also found provenance effects on colonized ECM root tip abundance and the community composition of ECM fungi. The relative geographic proximity of the Holden Arboretum to the origin location of Michigan trees relative to Maine trees may indicate some degree of local adaptation between sympatric tree genotypes and extant soil fungi. Further research is needed into local adaptation of ECM fungi with host genotypes and how this may interact to affect regional differences in plant responses to pests and pathogens.

## Figures and Tables

**Table 1:** Metadata on sampled trees from the Holden Arboretum Lower Baldwin Beech Orchard in fall 2020 and spring 2021. Symptomatology was categorized based on calculated blight scores from September 2020.

Fall Collection Date	Spring Collection Date	Tree ID	Provenance	Symptomatology
16 October 2020	15 June 2021	57-C	Michigan	Intermediate
16 October 2020	28 June 2021	57-G	Michigan	Intermediate
1 October 2020	29 June 2021	57-L	Michigan	Intermediate
1 October 2020	14 June 2021	57-M	Michigan	High
16 October 2020	29 June 2021	57-N	Michigan	Low
16 October 2020	29 June 2021	57-Q	Michigan	Low
1 October 2020	15 June 2021	57-S1	Michigan	Intermediate
16 October 2020	14 June 2021	58-A	Michigan	Intermediate
1 October 2020	29 June 2021	58-D	Michigan	Low
16 October 2020	15 June 2021	58-E	Michigan	Low
16 October 2020	15 June 2021	59-A	Michigan	Intermediate
NA	14 June 2021	61-C	Maine	Low
NA	29 June 2021	61-D	Maine	Low
16 October 2020	29 June 2021	61-G	Maine	Intermediate
NA	17 June 2021	61-I	Maine	Low
1 October 2020	14 June 2021	61-K	Maine	High
1 October 2020	14 June 2021	61-N	Maine	High
1 October 2020	14 June 2021	61-P	Maine	High
1 October 2020	14 June 2021	61-Q	Maine	High
NA	28 June 2021	61-U	Maine	Intermediate
16 October 2020	15 June 2021	94-A	Michigan	Intermediate
16 October 2020	28 June 2021	94-B	Michigan	Intermediate
1 October 2020	15 June 2021	94-E	Michigan	Intermediate
16 October 2020	15 June 2021	94-F	Michigan	Low
16 October 2020	14 June 2021	94-H	Michigan	Low
1 October 2020	29 June 2021	94-I	Michigan	Intermediate
1 October 2020	15 June 2021	94-J	Michigan	High
1 October 2020	14 June 2021	94-K	Michigan	High
1 October 2020	15 June 2021	94-M	Michigan	Intermediate
1 October 2020	29 June 2021	94-N	Michigan	Intermediate

**Table 2:** Mean abundance of ECM root tips from fall 2020, spring 2021, and the combined collections at varying tree conditions of BLD. The total numbers of colonized root tips were standardized per 1 g of dried root biomass. Tree condition was determined by two independent observers. Root tip abundance varied significantly between tree conditions in the fall 2020 collection.

Fall 2020 Collection (*N* = 26)			
Tree Condition	High Symptom (n = 7)	Intermediate Symptom (n = 13)	Low Symptom (n = 6)
ECM tips/g	188.6	388.7	549.8
Spring 2021 Collection (*N* = 30)			
Tree Condition	High Symptom (n = 7)	Intermediate Symptom (n = 14)	Low Symptom (n = 9)
ECM tips/g	254.2	271.8	267.8
Combined Fall 2020 and Spring 2021 Collections (*N* = 56)			
Tree Condition	High Symptom (n = 14)	Intermediate Symptom (n = 27)	Low Symptom (n = 15)
ECM tips/g	221.4	328.1	380.6

**Table 3:** GLS results (gls(log(Standardized Tip Abundance)~Condition)) of root tip abundance across trees of various BLD conditions for each collection and for combined collections data. Mean root tip abundances per gram of dried root mass varied significantly between trees of low- and high-symptom severity in the fall 2020 collection.

Coefficient	Value	Std. Error	*p* Value
Fall 2020 Collection			
Low Symptom	1.10	0.48	*p* = 0.03 *
Intermediate Symptom	0.74	0.40	*p* = 0.07
Spring 2021 Collection			
Low Symptom	0.16	0.43	*p* = 0.71
Intermediate Symptom	0.11	0.39	*p* = 0.78
Combined Fall 2020 and Spring 2021 Collection			
Low Symptom	0.56	0.32	*p* = 0.08
Intermediate Symptom	0.42	0.28	*p* = 0.15

* Mean abundance varied significantly from that of high-symptomatology trees at α = 0.05.

**Table 4:** Mean abundance of ECM root tips from fall 2020, spring 2021, and combined collections in the different provenances. The total numbers of colonized root tips were standardized per 1 g of dried root biomass. Tree condition was determined by two independent observers. Root tip abundance varied significantly between Maine and Michigan when collections were combined.

Fall 2020 Collection (*N* = 26)		
Provenance	Maine (n = 5)	Michigan (n = 21)
ECM tips/g	244.32	402.4
Spring 2021 Collection (*N* = 30)		
Provenance	Maine (n = 9)	Michigan (n = 21)
ECM tips/g	114.7	331.5
Combined Fall 2020 and Spring 2021 Collection (*N* = 56)		
Provenance	Maine (n = 14)	Michigan (n = 42)
ECM tips/g	161.01	366.96

**Table 5:** GLS results (gls(log(Standardized Tip Abundance)~Provenance)) of root tip abundance across trees of different provenance for each collection and for combined collections data. Mean root tip abundances per gram of dry root mass varied significantly between Maine and Michigan trees when collections data were combined and in the spring 2021 collection alone.

Coefficient	Value	Std. Error	*p* Value
Fall 2020 Collection			
Michigan	0.36	0.46	*p* = 0.44
Spring 2021 Collection			
Michigan	1.02	0.27	*p* < 0.001 *
Combined Fall 2020 and Spring 2021 Collection			
Michigan	0.78	0.25	*p* = 0.003 *

* Mean abundance varied significantly from Maine trees at α = 0.05.

## Data Availability

Not applicable.

## Supplementary Materials

The following supporting information can be downloaded at: https://www.mdpi.com/article/10.3390/jof9040497/s1, Figure S1: Site map of USFS Beech Research; Figure S2: Examples of a High (Tree ID 57-M; panels a–c) and Intermediate (Tree ID 59-A; panels d–f) symptomatology tree; Table S1: Ranking system for determining a quantitative symptom severity score; Table S2: Results of Wald Tests showing zOTUs that were significantly affected by tree condition (high vs. low and intermediate vs. low symptomatology trees were compared) or tree source.

## References

[1] Ewing C.J., Hausman C.E., Pogacnik J., Slot J., Bonello P. (2018). Beech leaf disease: An emerging forest epidemic. For. Pathol..

[2] Carta L., Handoo Z., Li S., Burke D. (2020). Beech leaf disease symptoms caused by newly recognized nematode subspecies *Litylenchus crenatae mccannii* (Anguinata) described from *Fagus grandifolia* in North America. For. Pathol..

[3] Burke D., Hoke A., Koch J. (2020). The emergence of beech leaf disease in Ohio: Probing the plant microbiome in search of the cause. For. Pathol..

[4] Bose A.K., Weiskittel A., Wagner R.G. (2017). A three-decade assessment of climate-associated changes in forest composition across the north-eastern USA. J. Appl. Ecol..

[5] Reed S.E., Volk D., Martin D.K.H., Hausman C.E., Macy T., Tomon T., Cousins S. (2023). The distribution of beech leaf disease and the causal agents of beech bark disease (Cryptoccocus fagisuga, Neonectria faginata, N. ditissima) in forests surrounding Lake Erie and future implications. For. Ecol. Manag..

[6] Finlay R.D. (2008). Ecological aspects of mycorrhizal symbiosis: With special emphasis on the functional diversity of interactions involving the extraradical mycelium. J. Exp. Bot..

[7] Martin F., Kohler A., Murat C., Veneault-Fourrey C., Hibbett D.S. (2016). Unearthing the roots of ectomycorrhizal symbioses. Nat. Rev. Microbiol..

[8] Tresender K., Torn M., Masiello C. (2006). An ecosystem-scale radiocarbon tracer to test use of litter carbon by ectomycorrhizal fungi. Soil Biol. Biochem..

[9] Burke D.J., Smemo K.A., Hewins C.R. (2014). Ectomycorrhizal fungi isolated from old-growth northern hardwood forest display variability in extracellular enzyme activity in the presence of plant litter. Soil Biol. Biochem..

[10] Vrålstad T., Schumacher T., Taylor A.F.S. (2002). Mycorrhizal synthesis between fungal strains of the *Hymenoscyphus ericae* aggregate and potential ectomycorrhizal and ericoid hosts. New Phytol..

[11] Peterson R.L., Massicotte H.B. (2004). Exploring structural definitions of mycorrhizas, with emphasis on nutrient-exchange interfaces. Can. J. Bot..

[12] Luo Z.-B., Janz D., Jiang X., Gobel C., Wildhagen H., Tan Y., Rennenberg H., Feussner I., Polle A. (2009). Upgrading Root Physiology for Stress Tolerance by Ectomycorrhizas: Insights from Metabolite and Transcriptional Profiling into Reprogramming for Stress Anticipation. Plant Physiol..

[13] Wang J., Zhang H., Gao J., Zhang Y., Liu Y., Tang M. (2021). Effects of ectomycorrhizal fungi (Suillus variegatus) on the growth, hydraulic function, and non-structural carbohydrates of Pinus tabulaeformis under drought stress. BMC Plant Biol..

[14] Chu H., Wang C., Li Z., Wang H., Xiao Y., Chen J., Tang M. (2019). The dark septate endophytes and ectomycorrhizal fungi effect on *Pinus tabulaeformis* Carr. seedling growth and their potential effects to pine wilt disease resistance. Forests.

[15] Gehring C.A., Whitham T.G. (1994). Comparisons of ectomycorrhizae on pinyon pines (*Pinus edulis*; Pinaceae) across extremes of soil type and herbivory. Am. J. Bot..

[16] Gehring C.A., Whitham T.G. (2002). Mycorrhizae-Herbivore Interactions: Population and Community Consequences. Mycorrhizal Ecology.

[17] Vendettuoli J.F., Orwig D.A., Krumins J.A., Waterhouse M.D., Preisser E.L. (2015). Hemlock woolly adelgid alters fine root bacterial abundance and mycorrhizal associations in eastern hemlock. For. Ecol. Manag..

[18] Lewis J.D., Licitra J., Tuininga A.R., Sirulnik A., Turner G.D., Johnson J. (2008). Oak seedling growth and ectomycorrhizal colonization are less in eastern hemlock stands infested with hemlock woolly adelgid than in adjacent oak stands. Tree Physiol..

[19] Chu H., Wang C., Wang H., Chen H., Tang M. (2016). Pine wilt disease alters soil properties and root-associated fungal communities in *Pinus tabulaeformis* forest. Plant Soil.

[20] Chu H., Tang M., Wang H., Wang C. (2018). Pinewood nematode infection alters root mycoflora of *Pinus tabulaeformis* Carr. J. Appl. Microbiol..

[21] Cale J.A., Garrison-Johnston M.T., Teale S.A., Castello J.D. (2017). Beech bark disease in North America: Over a century of research revisited. For. Ecol. Manag..

[22] Pena R., Offermann C., Simon J., Naumann P.S., Geßler A., Holst J., Dannenmann M., Mayer H., Kögel-Knabner I., Rennenberg H. (2010). Girdling Affects Ectomycorrhizal Fungal (EMF) Diversity and Reveals Functional Differences in EMF Community Composition in a Beech Forest. Appl. Environ. Microbiol..

[23] Courty P.-E., Buèe M., Diedhiou A.G., Frey-Klett P., Le Tacon F., Rineau F., Turpault M.-P., Uroz S., Garbaye J. (2010). The role of ectomycorrhizal communities in forest ecosystem processes: New perspectives and emerging concepts. Soil Biol. Biochem..

[24] Agerer R. (2001). Exploration types of ectomycorrhizae: A proposal to classify ectomycorrhizal mycelial systems according to their patterns of differentiation and putative ecological importance. Mycorrhiza.

[25] Lilleskov E.A., Hobbie E.A., Horton T.R. (2011). Conservation of ectomycorrhizal fungi: Exploring the linkages between functional and taxonomic responses to anthropogenic N deposition. Fungal Ecol..

[26] Johnson N.C., Wilson G.W.T., Bowker M.A., Wilson J.A., Miller R.M. (2010). Resource limitation is a driver of local adaptation in mycorrhizal symbioses. Proc. Natl. Acad. Sci. USA.

[27] Rúa M.A., Antoninka A., Antunes P.M., Chaudhary V.B., Gehring C., Lamit L.J., Piculell B.J., Bever J.D., Zabinski C., Meadow J.F. (2016). Home-field advantage? Evidence of local adaptation among plants, soil, and arbuscular mycorrhizal fungi through meta-analysis. BMC Evol. Biol..

[28] Koch J.L. (2010). Beech bark disease: The oldest “new” threat to American beech in the United States. Outlooks Pest Manag..

[29] Koch J., Allmaras M., Barnes S., Berrang P., Hall T., Iskra A., Kochenderfer J., Macdonald W., Rogers S., Rose J., Potter K.M., Conkling B.L. (2015). Beech seed orchard development: Identification and propagation of beech bark resistant American beech trees. Forest Health Monitoring: National Status, Trends and Analysis.

[30] Burke D.J. (2008). Effects of *Alliaria petiolata* (garlic mustard: Brassicaceae) on mycorrhizal colonization and community structure in three herbaceous plants in a mixed deciduous forest. Am. J. Bot..

[31] Martin K.J., Rygiewicz P.T. (2005). Fungal-specific PCR primers developed for analysis of the ITS region of environmental DNA extracts. BMC Microbiol..

[32] Yang R.-H., Su J.-H., Shang J.-J., Wu Y.-Y., Li Y., Bao D.-P., Yao Y.-J. (2018). Evaluation of the ribosomal DNA internal transcribed spacer (ITS), specifically ITS1 and ITS2, for the analysis of fungal diversity by deep sequencing. PLoS ONE.

[33] Edgar R.C. (2016). UNOISE2: Improved error-correction for Illumina 16S and ITS amplicon sequencing. bioRxiv.

[34] Edgar R.C. (2010). Search and clustering orders of magnitude faster than BLAST. Bioinformatics.

[35] Martin M. (2011). Cutadapt removes adapter sequences from high-throughput sequencing reads. EMBnet.J..

[36] Edgar R.C. (2016). SINTAX: A simple non-Bayesian taxonomy classifier for 16S and ITS sequences. bioRxiv.

[37] UNITE Community (2019). UNITE USEARCH/UTAX Release for Fungi.

[38] R Development Core Team (2020). A Language and Environment for Statistical Computing.

[39] Pinheiro J., Bates D., DebRoy S., Sarkar D., R Core Team (2020). _nlme: Linear and Nonlinear Mixed Effects Models_. R Package Version 3.1-150. https://CRAN.R-project.org/package=nlme.

[40] Wickham H. (2016). Ggplot2: Elegant Graphics for Data Analysis.

[41] McMurdie P.J., Holmes S. (2013). Phyloseq: An R package for reproducible interactive analysis and graphics of microbiome census data. PLoS ONE.

[42] Oksanen J., Blanchet F.G., Friendly M., Kindt R., Legendre P., McGlinn D., Minchin P.R., O’Hara R.B., Simpson G.L., Solymos P. (2019). R Package Version 2.5-6. https://CRAN.R-project.org/package=vegan.

[43] Love M.I., Huber W., Anders S. (2014). Moderated estimation of fold change and dispersion for RNA-seq data with DESeq2. Genome Biol..

[44] Zhu A., Ibrahim J.G., Love M.I. (2018). Heavy-tailed prior distributions for sequence count data: Removing the noise and preserving large differences. Bioinformatics.

[45] Nguyen N.H., Song Z., Bates S.T., Branco S., Tedersoo L., Menke J., Schilling J.S., Kennedy P.G. (2016). FUNGuild: An open annotation tool for parsing fungal community datasets by ecological guild. Fungal Ecol..

[46] Smith S.E., Read D.J. (2008). Mycorrhizal Symbiosis.

[47] Nabity P.D., Zavala J.A., DeLucia E.A. (2009). Indirect suppression of photosynthesis on individual leaves by arthropod herbivory. Ann. Bot..

[48] Welsh A., Burke D., Hamerlynck E., Hahn D. (2009). Seasonal analyses of arbuscular mycorrhizae, nitrogen-fixing bacteria and growth performance of the salt marsh grass *Spartina patens*. Plant Soil..

[49] Hewins C., Carrino-Kyker S., Burke D. (2015). Seasonal variation in mycorrhizal fungi colonizing roots of *Allium tricoccum* (wild leek) in a mature mixed hardwood forest. Mycorrhiza.

[50] Tierney G.L., Fahey T.J., Groffman P.M., Hardy J.P., Fitzhugh R.D., Driscoll C.T., Yavitt J.B. (2003). Environmental control of fine root dynamics in a northern hardwood forest. Global Chang. Biol..

[51] Swaty R.L., Deckert R.J., Whitham T.G., Gehring C.A. (2004). Ectomycorrhizal abundance and community composition shifts with drought: Predictions from tree rings. Ecology.

[52] Burke D., Carrino-Kyker S., Burns J. (2019). Is it climate or chemistry? Soil fungal communities respond to soil nutrients in a multi-year high-resolution analysis. Ecosphere.

[53] Carrino-Kyker S., Coyle K., Kluber L., Burke D. (2019). Fungal and Bacterial Communities Exhibit Consistent Responses to Reversal of Soil Acidification and Phosphorus Limitation over Time. Microorganisms.

[54] Rubini A., Riccioni C., Belfiori B., Paolocci F. (2014). Impact of the competition between mating types on the cultivation of *Tuber melanosporum*: Romeo and Juliet and the matter of space and time. Mycorrhiza.

[55] Pánková H., Münzbergová Z., Rydlová J., Vosátka M. (2014). Co-Adaptation of Plants and Communities of Arbuscular Mycorrhizal Fungi to Their Soil Conditions. Folia Geobot..

[56] Hoeksema J.D., Hernandez J.V., Rogers D.L., Mendoza L.L., Thompson J.N. (2012). Geographic divergence in a species-rich symbiosis: Interactions between Monterey pines and ectomycorrhizal fungi. Ecology.

[57] Rúa M.A., Lamit L.J., Gehring C., Antunes P.M., Hoeksema J.D., Zabinski C., Karst J., Burns C., Woods M. (2018). Accounting for local adaptation in ectomycorrhizas: A call to track geographical origin of plants, fungi, and soils in experiments. Mycorrhiza.

[58] Schelkle M., Peterson R.L. (1996). Suppression of common root pathogens by helper bacteria and ectomycorrhizal fungi in vitro. Mycorrhiza.

[59] Kanekar S.S., Cale J.A., Erbilgin N. (2018). Ectomycorrhizal fungal species differentially affect the induced defensive chemistry of lodgepole pine. Oecologia.

[60] Vishwanathan K., Zienkiewicz K., Liu Y., Janz D., Feussner I., Polle A., Haney C.H. (2020). Ectomycorrhizal fungi induce systemic resistance against insects on a nonmycorrhizal plant in a CERK1-dependent manner. New Phytol..

[61] Dreischhoff S., Das I.S., Jakobi M., Kasper K., Polle A. (2020). Local responses and systemic induced resistance mediated by ectomycorrhizal fungi. Front. Plant Sci..

[62] Chu H., Wang H., Zhang Y., Li Z., Wang C., Dai D., Tang M. (2021). Inoculation with ectomycorrhizal fungi and dark septate endophytes contributes to the resistance of *Pinus* spp. to pine wilt disease. Front. Microbiol..

[63] Costanza R., d’Arge R., de Groot R., Farber S., Grasso M., Hannon B., Limburg K., Naeem S., O’Niell R.V., Paruelo J. (1997). The value of the world’s ecosystem services and natural capital. Nature.

[64] Ferlian O., Goldmann K., Eisenhauer N., Tarkka M.T., Buscot F., Heintz-Buschart A. (2021). Distinct effects of host and neighbour tree identity on arbuscular and ectomycorrhizal fungi along a tree diversity gradient. ISME Commun..

