# Supplementary material for: Beech Leaf Disease Severity Affects Ectomycorrhizal Colonization and Fungal Taxa Composition
Source: J Fungi (Basel). 2023 Apr 21;9(4):497. doi: 10.3390/jof9040497 (PMC10146144; doi:10.3390/jof9040497)
Supplement: Supplementary file 1 [file jof-09-00497-s001.zip › jof-2331936-supplementary.docx]

**Supplemental Figure 1.** Site map of USFS Beech Research Plot (Koch 2010), at the Holden Arboretum. Symptomatology is indicated by color. Trees sampled in both fall 2020 and spring 2021 are circled with solid line, and trees added to the spring 2021 sampling are circled in dashed lines. All trees accessioned into the USFS Beech Research Plot were from genetic families that showed resistance to Beech Bark Disease. The total size of the plantation is 3.73 acres.


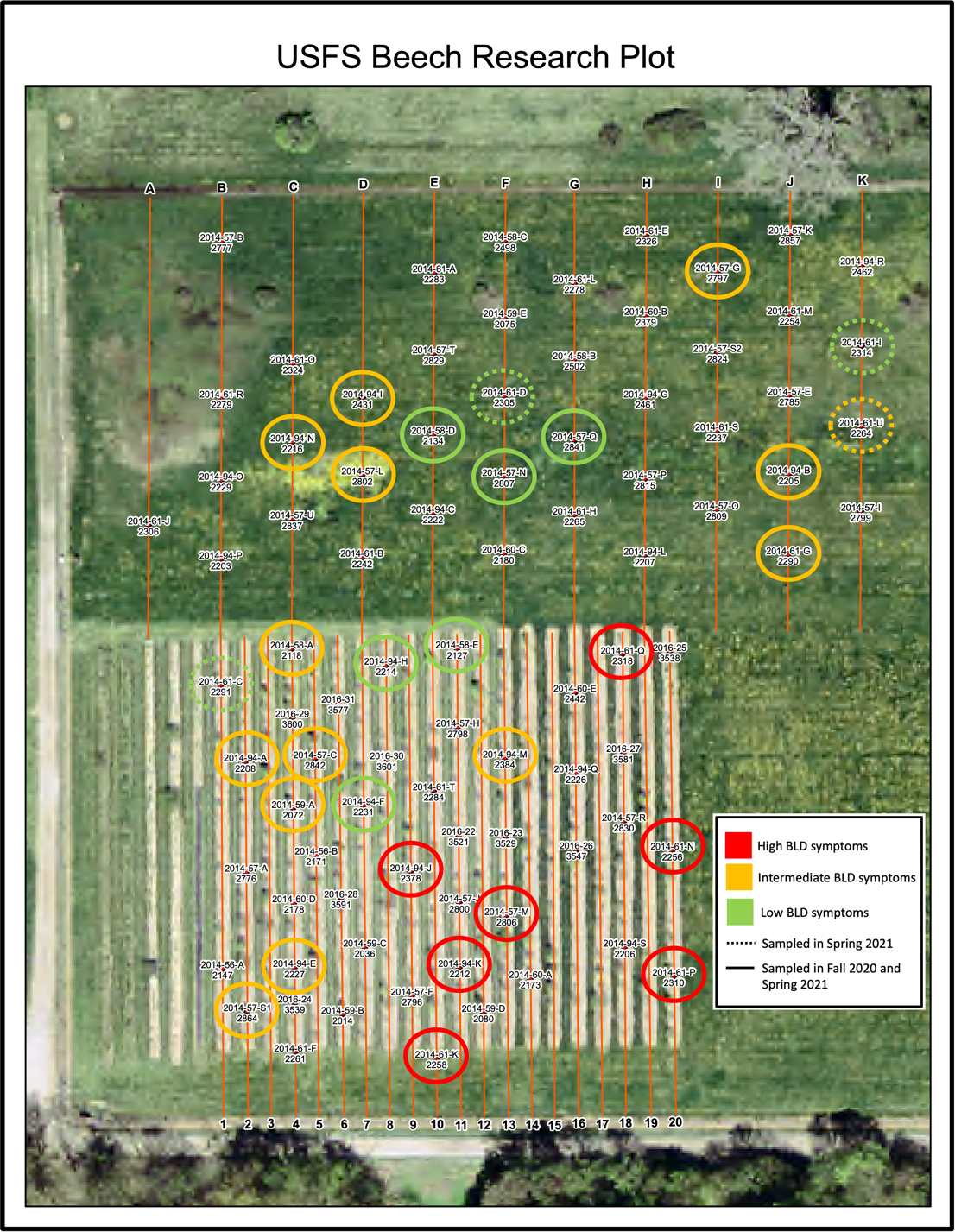


**Supplemental Table 1.** Ranking system for determining a quantitative symptom severity score. Rankings were applied to each tree in the study in September 2020. Ratings were based on a calculated symptom severity score. Trees were ranked by two observers, each of whom estimated percent of symptomatic leaves at three locations around the tree. Rankings were given a numerical value based on these percentages. Calculated values were averaged to a whole number and were used to determine the ultimate symptomatology category of the tree (high, intermediate, and low symptomatology).

| Symptomatology Ranking | 1 | 2 | 3 | 4 | 5 |
| --- | --- | --- | --- | --- | --- |
| BLD Percent Cover | <10 | 10 to 25 | 26 to 50 | 51 to 75 | >75 |

**
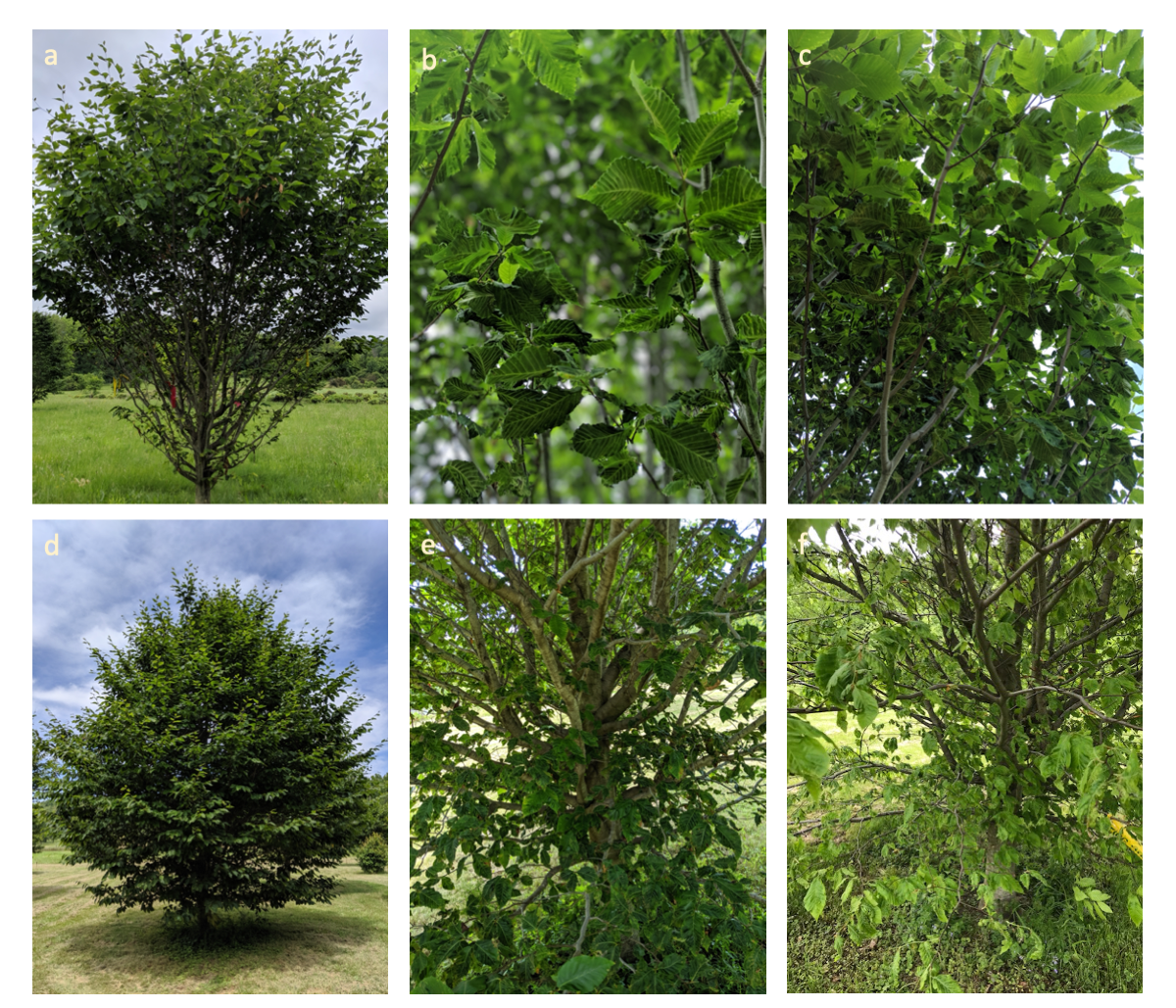
**

**Supplemental Figure 2.** Examples of a High (Tree ID 57-M; panels a-c) and Intermediate (Tree ID 59-A; panels d-f) symptomatology tree. Pictures were taken between 22 May and 19 July of 2019.

**Supplemental Table 2.** Results of Wald Tests showing zOTUs that were significantly affected by tree condition (high vs. low and intermediate vs. low symptomatology trees were compared) or tree source.

| zOTU | LFC^a^ shrunk | LFC SE^b^ shrunk | Adjusted p-value^c^ | Phylum | Class | Order | Family | Genus | Guild^d^ |
| --- | --- | --- | --- | --- | --- | --- | --- | --- | --- |
| High vs. Low Symptamotology (A positive LFC value indicates greater normalized abundance in High symptom trees) | | | | | | | | | |
| zOTU19 | -3.50e-07 | 0.0014 | 1.83e-17 | Basidiomycota | Agaricomycetes | Agaricales |  |  |  |
| zOTU51 | 2.57e-07 | 0.0014 | 4.41e-07 | Ascomycota | Pezizomycetes | Pezizales | Tuberaceae | Tuber | Ectomycorrhizal |
| zOTU66 | 3.31 | 1.071 | 0.0040 |  |  |  |  |  |  |
| zOTU34 | 7.27e-07 | 0.0014 | 0.0040 | Basidiomycota | Agaricomycetes | Russulales | Russulaceae | Russula | Ectomycorrhizal |
| zOTU70 | -3.22e-06 | 0.0014 | 0.0040 | Ascomycota | Eurotiomycetes | Chaetothyriales | Herpotrichiellaceae |  | Animal Pathogen- Fungal Parasite- Undefined Saprotroph |
| zOTU15 | 1.57e-07 | 0.0014 | 0.0092 | Basidiomycota | Agaricomycetes | Agaricales | Cortinariaceae | Cortinarius | Ectomycorrhizal |
| zOTU4 | 4.10e-07 | 0.0014 | 0.010 | Basidiomycota | Agaricomycetes | Agaricales | Cortinariaceae | Cortinarius | Ectomycorrhizal |
| zOTU56 | 9.83e-07 | 0.0014 | 0.027 | Basidiomycota | Agaricomycetes | Russulales | Russulaceae | Lactarius | Ectomycorrhizal |
| zOTU153 | 1.30e-06 | 0.0014 | 0.027 | Ascomycota | Sordariomycetes | Hypocreales | Nectriaceae |  | Animal Pathogen-Endophyte- Lichen Parasite- Plant Pathogen- Soil Saprotroph- Wood Saprotroph* |
| zOTU42 | 1.76 | 2.0093 | 0.049 |  |  |  |  |  |  |
| zOTU12 | 3.98e-07 | 0.0014 | 0.049 | Ascomycota | Pezizomycetes | Pezizales | Tuberaceae | Tuber | Ectomycorrhizal |
| zOTU78 | 4.56e-07 | 0.0014 | 0.050 | Basidiomycota | Agaricomycetes | Agaricales |  |  |  |
| Intermediate vs. Low Symptamotology (A positive LFC value indicates greater normalized abundance in Intermediate symptom trees) | | | | | | | | | |
| zOTU39 | 2.88e-07 | 0.0014 | 4.84e-23 | Ascomycota | Pezizomycetes | Pezizales | Tuberaceae | Tuber | Ectomycorrhizal |
| zOTU34 | 5.54 | 1.81 | 1.47e-10 | Basidiomycota | Agaricomycetes | Russulales | Russulaceae | Russula | Ectomycorrhizal |
| zOTU49 | 11.73 | 2.90 | 1.62e-06 | Basidiomycota | Agaricomycetes | Russulales | Russulaceae | Russula | Ectomycorrhizal |
| zOTU4 | 4.16e-07 | 0.0014 | 0.00086 | Basidiomycota | Agaricomycetes | Agaricales | Cortinariaceae | Cortinarius | Ectomycorrhizal |
| zOTU214 | 1.60e-06 | 0.0014 | 0.0028 |  |  |  |  |  |  |
| zOTU12 | 3.88e-07 | 0.0014 | 0.0028 | Ascomycota | Pezizomycetes | Pezizales | Tuberaceae | Tuber | Ectomycorrhizal |
| zOTU104 | 2.34 | 0.99 | 0.0044 | Ascomycota | Eurotiomycetes | Chaetothyriales | Herpotrichiellaceae | Minimelanolocus | Animal Pathogen- Fungal Parasite- Undefined Saprotroph |
| zOTU117 | 2.046 | 0.92 | 0.012 | Ascomycota |  |  |  |  |  |
| zOTU13 | 4.14e-07 | 0.0014 | 0.014 | Ascomycota | Pezizomycetes | Pezizales | Tuberaceae | Tuber | Ectomycorrhizal |
| zOTU54 | 1.44 | 0.86 | 0.018 | Ascomycota | Eurotiomycetes | Chaetothyriales |  |  |  |
| zOTU209 | 1.77e-06 | 0.0014 | 0.034 | Ascomycota | Sordariomycetes | Hypocreales | Nectriaceae | Fusarium | Animal Pathogen-Endophyte- Lichen Parasite- Plant Pathogen- Soil Saprotroph- Wood Saprotroph* |
| zOTU10 | 1.49e-06 | 0.0014 | 0.037 | Basidiomycota | Agaricomycetes | Russulales | Russulaceae | Russula | Ectomycorrhizal |
| zOTU100 | 1.20 | 1.028 | 0.037 | Ascomycota |  |  |  |  |  |
| zOTU17 | 9.56e-07 | 0.0014 | 0.041 | Basidiomycota | Agaricomycetes | Russulales | Russulaceae | Russula | Ectomycorrhizal |
| zOTU36 | 1.20e-07 | 0.0014 | 0.042 | Basidiomycota | Agaricomycetes | Agaricales | Hymenogastraceae | Hymenogaster | Ectomycorrhizal* |
| zOTU15 | 2.19e-07 | 0.0014 | 0.047 | Basidiomycota | Agaricomycetes | Agaricales | Cortinariaceae | Cortinarius | Ectomycorrhizal |
| Michigan vs. Maine (A positive LFC value indicates greater normalized abundance in the Michigan provenance) | | | | | | | | | |
| zOTU19 | 1.49 | 0.99 | 0.00030 | Basidiomycota | Agaricomycetes | Agaricales |  |  |  |
| zOTU56 | 3.20 | 0.95 | 0.00030 | Basidiomycota | Agaricomycetes | Russulales | Russulaceae | Lactarius | Ectomycorrhizal |
| zOTU12 | 1.44 | 0.99 | 0.00030 | Ascomycota | Pezizomycetes | Pezizales | Tuberaceae | Tuber | Ectomycorrhizal |
| zOTU104 | 2.54 | 0.66 | 0.0043 | Ascomycota | Eurotiomycetes | Chaetothyriales | Herpotrichiellaceae | Minimelanolocus | Animal Pathogen- Fungal Parasite- Undefined Saprotroph |
| zOTU158 | 2.82 | 0.76 | 0.0043 | Mortierellomycota | Mortierellomycetes | Mortierellales | Mortierellaceae | Mortierella | Endophyte- Litter Saprotroph- Soil Saprotroph- Undefined Saprotroph |
| zOTU6 | 1.75 | 1.00075 | 0.0043 | Basidiomycota | Agaricomycetes | Agaricales | Cortinariaceae | Cortinarius | Ectomycorrhizal |
| zOTU54 | 0.55 | 0.52 | 0.0065 | Ascomycota | Eurotiomycetes | Chaetothyriales |  |  |  |
| zOTU285 | 3.08 | 1.86e-06 | 0.0072 | Basidiomycota | Agaricomycetes | Agaricales | Omphalotaceae | Gymnopus | Wood Saprotroph |
| zOTU177 | 2.50 | 0.93 | 0.014 | Basidiomycota |  |  |  |  |  |
| zOTU2 | 0.48 | 0.93 | 0.014 | Ascomycota | Pezizomycetes | Pezizales | Tuberaceae | Tuber | Ectomycorrhizal |
| zOTU25 | 1.046 | 0.96 | 0.014 | Ascomycota | Pezizomycetes | Pezizales | Tuberaceae | Tuber | Ectomycorrhizal |
| zOTU222 | 2.45 | 0.79 | 0.018 |  |  |  |  |  |  |
| zOTU15 | 0.71 | 0.89 | 0.021 | Basidiomycota | Agaricomycetes | Agaricales | Cortinariaceae | Cortinarius | Ectomycorrhizal |
| zOTU27 | 1.54 | 0.71 | 0.043 | Basidiomycota | Agaricomycetes | Agaricales |  |  |  |
| zOTU13 | -3.41 | 1.00 | 0.048 | Ascomycota | Pezizomycetes | Pezizales | Tuberaceae | Tuber | Ectomycorrhizal |

^a^ log_2_ fold change values shrunken with the apeglm method in *DESeq2*.

^b^Standard error of the shrunken LFC values.

^c^p-values adjusted with the Benjamini-Hochberg false discovery rate.

^d^Guilds with a confidence ranking of “possible” are marked with an asterisk. All other guilds had at a “probable” or “highly probably” confidence ranking.
